# A Modified Intraoral Resin Mouthguard to Prevent Self-Mutilations in Lesch-Nyhan Patients

**DOI:** 10.1155/2014/396830

**Published:** 2014-07-02

**Authors:** Giulia Ragazzini, Alessia Delucchi, Enrico Calcagno, Roberto Servetto, Gloria Denotti

**Affiliations:** Scuola di Specializzazione in Ortognatodonzia, Università degli Studi di Cagliari, UOC Odontoiatria Istituto Giannina Gaslini, Padiglione 20, Via Gerolamo Gaslini 5, 16147 Genova, Italy

## Abstract

Lesch-Nyhan syndrome, described in 1964 by Lesch and Nyhan, is a X-linked recessive disorder, occurring in 1 : 100000 to 1 : 380000 live births. LNS is characterized by a decrease in activity of hypoxanthine guanine phosphoribosyl transferase, an enzyme involved in purine metabolism, resulting in overproduction of uric acid. Hyperuricemia and neurological features including choreoathetoid spasticity, self-mutilation, and mental retardation clinically characterize this syndrome. In LNS patients the typical feature is loss of tissue from biting themselves with partial or complete amputation of fingers, lips, and tongue. The self-mutilation compares with the eruption of the deciduous teeth. Several drugs trials have been administered to improve self-destructive behavior and invasive treatment approaches, such as extractions of teeth and orthognathic surgery, have been suggested with variable effectiveness. Nowadays prevention is, therefore, the standard of care. The role of dentistry is essential in the management of the self-mutilating behavior, because the teeth represent the main self-injury instrument. This report presents a revision of various therapeutic approaches to manage self-destruction, highlighting the effectiveness of a preventive treatment. It describes a new technique: a resin mouthguard, realized at Gaslini Hospital, to obtain immediate healing of the oral lesions, confirmed in the follow-up period.

## 1. Introduction

Lesch-Nyhan syndrome was first recognized and clinically characterized in 1964 by Lesch and Nyhan [1], observing two affected brothers. It occurs in 1 : 100000 to 380000 male live births. LNS is a rare X-linked recessive disorder, resulting from a mutation on the gene encoding for the enzyme hypoxanthine-guanine phosphoribosyltransferase (HGPRT), located on the long arm of the X chromosome between Xq26 and Xq27 [2].

The HGPRT is an enzyme involved in purine metabolism, which catalyses the condensation reaction which combines phosphoribosyl pyrophosphate (PRPP) and the purine bases hypoxanthine and guanine to form the respective nucleotides, inosinic acid, and guanylin acid.

The HGPRT deficiency causes a backlog of guanine and hypoxanthine that oxidize in uric acid and an increase of intracellular PRPP with a consequent major production of new purine. It results in an overproduction and accumulation of uric acid, which, if untreated, usually leads to renal failure and death in early childhood.

One of the first symptoms of the disease is the presence of orange sand-like crystals of uric acid in the diapers of the affected infant. Overproduction of uric acid may lead to the development of uric acid crystals or stones in the kidneys (nephrolithiasis), ureters, or bladder, causing hematuria and increasing the risk of urinary tract infection, of nephrite, and of renal failure. Another potential consequence of untreated hyperuricemia is gouty arthritis caused by uric acid crystal deposition in articular cartilage.

Individuals with Lesch-Nyhan syndrome typically have a normal prenatal and perinatal course. The affected children appear to be normal for the first few months of life, but hypotonia and development delay are evident from age three to six months. Later extrapyramidal involvement with dystonia, choreoathetosis, opisthotonos, dysarthria, dysphagia, and sometimes ballismus becomes evident. The patients also develop signs of pyramidal involvement as spasticity, hyperreflexia, and extensor plantar reflexes. The motor disability is so extensive that most individuals never walk and are confined to a wheelchair. The cognitive disturbs, attention deficit problems, and mental retardation are also present in patients with Lesch-Nyhan syndrome [3].

The most distinctive symptom is the compulsive self-injury behavior. The affected children appear normal for the first months of life; with the eruption of deciduous teeth, they begin to show self-mutilative behavior, biting their oral/perioral tissue and their fingers. Later patients mutilate themselves not only with biting but also with their fingers. The finger nails may be completely ripped, and the self-destructive process involves the bone. In some cases, extensive lesions appear as a mixture of trauma and local infection. The secondary infection complicates and retards the healing of traumatic lesions [4, 5].

Nyhan (1997) reported that children with LNS feel pain and remorse when they mutilate themselves, but they are unable to control and to stop their action. They are usually relaxed when they have physical restraints, instead when it is removed the patients become agitated and their compulsive actions increase [6].

The relationship between the enzyme deficiency and the neurological manifestations in Lesch-Nyhan syndrome is not so clear. Those disorders may be associated with deficits in dopaminergic activity in the basal ganglia; neurochemical studies of LNS patients' brains show large reductions in DA levels, elevated numbers of DA receptors, and decreased levels of DA transporters [7].

The therapy with allopurinol, a xanthine oxidase inhibitor which blocks the metabolism of hypoxanthine and xanthine into uric acid, can control the overproduction of uric acid, reducing the risk of nephrolithiasis, gouty arthritis, and correlated diseases. The dose of allopurinol has to maintain the uric acid within normal limits; an excessive allopurinol therapy results in the accumulation of hypoxanthine and xanthine, which could result in xanthine stones.

The pharmacological treatment of self-injurious behavior is addressed to control the deregulation in the dopaminergic, opiatergic, or serotonergic systems, as a possible cause of compulsive self-mutilating behavior. The administration of baclofen or benzodiazepines helps in muscle relaxation and keeps the patient calm. The use of hydroxytryptophan together with decarboxylase inhibitor has been proven effective in reducing self-mutilation for a short time. Recent therapeutic options also include gabapentin, botulinum toxin A, injected into bilateral masseters, and deep brain stimulation in globus pallidus.

The self-destruction behavior should be managed by a combination of physical restraints, behavior modifications, and pharmaceutical therapy, according to Olson and Houlihan 2000 [8].

## 2. Management of Self-Mutilative Behavior: Literature Review

In the literature various therapeutic approaches to manage self-destruction behavior are described, but to date the treatment to solve the source of the problem has not been found yet. Pharmacological treatment of dopaminergic dysfunction, the possible opiate, and/or serotonin system dysfunction have shown variable successes, but there are disadvantages to pharmacologic treatment, as it usually requires chronic use of the drug that often places the patients in a state of chronic stupor.

The use of botulinum toxin A (BTX-A) should be considered as a good treatment for self-mutilating behavior in LNS, but the action of BTX-A in decreasing self-inflicted injury is not clear. BTX-A may be acting directly on peripheral nerve endings, inhibiting the release of acetylcholine and indirectly on the basal ganglia, resulting in muscle weakness and in decreased behavior. Inquiries on BTX-A nowadays are not enough and a further study is necessary to define the mechanism of action and the therapeutic results [9, 10].

The “deep-brain stimulation” has been performed on a few patients with Lesch-Nyhan syndrome and some patients experienced a decrease in spastic self-injurious symptoms. Deep brain stimulation must be considered experimental at present.

Published literature suggests that the role of dentist is crucial to prevent and control self-mutilation [11]. In several cases, the extraction of deciduous teeth is adopted in young patient, but the extraction of all teeth is necessary also in permanent dentition.

The extraction of only anterior permanent teeth (incisors and canines), as recommended by Rosenbloom and other authors, is not effective in preventing self-injury and so all permanent teeth are involved. The extraction of all teeth represents an extreme solution that may cause functional problems and requires a treatment under general anesthesia. To avoid the extractions, a series of intraoral appliance is suggested to limit self-injures.

Budnick studying a Lesch-Nyhan syndrome patient, unable to chew his lips for lack of anterior occlusion, decided to create an acrylic splint firstly cemented in the lower arch and secondly in the upper one. He experimented a new technique by covering the posterior teeth to create anterior open bite, eliminating wounds caused by incisors [12]. Dicks highlighted the necessity of different therapeutic steps for different self-mutilative behaviors. In the most collaborative patients with few self-mutilations he proposed only normal care of preventive odontology; in patients showing such a grade of collaboration and of spasticity to allow the intraoral alginate impression, he suggested the use of soft splint without cemented retention which caused hygienic and demineralization problems. Finally, in patients with insufficient collaboration the only solution was represented by the extractions of all teeth [13]. A hard acrylic chin-cup with lip extension fitted by means of orthodontic headgear was proposed. The appliance was well tolerated by patients and it guaranteed the health of the oral tissue but increased the dribbling habit with a consequent fungal infection of chins dermis. To solve fungal inflammation the appliance had to be removed. The long use of this appliance was not possible [14]. A thin soft resin mouth guard was employed to prevent finger and lower lip self-mutilation in combination with an arm restraint. In six days of treatment and after three months the patient self-mutilations disappeared and he stopped biting himself, also without the mouthguard [15]. In order to prevent dermatitis a soft resin mouthguard not covering the lip was built. It was first cemented and then to increase the retention he preferred heat-cured acrylic resin due to adequate rigidity and he added head strap; it had to rebuild to be adapted to development of teeth and it was not indicated in periodontal patients [16]. In some resistant casese orthognathic surgery was proposed to create open bite and when the conservative options of treatment failed he suggested the amputation of teeth, as an alternative method to extraction of all teeth to preserve the alveolar bone. The upper and the lower incisors, canines, and the first premolars were performed with formocresol pulpotomies, the crowns were amputated on gingival level, and the dental root canals were filled with light curing glass ionomer cement and polished with softlex discs. The patient was sedated using choral hydrate and hydroxane, but in case of poor cooperation the general anesthesia was indicated [17–19]. A removable acrylic dental appliance was made to create anterior open bite, orthodontic acrylic resin covered the palate, and occlusal surface of posterior teeth and Adam's claps on first upper molar increased the retention [20].

A resin mouthguard thick in posterior part was combined with a lip bumper in order to open anterior bite and to protrude lower lip from the teeth, but this treatment had minimal to partial success [21]. The acrylic resin bite plate's retention was increased by a combination between Adam's clasps on the premolars and ball clasps among incisors [22].

## 3. Method

The operators of the orthodontic ward of Gaslini Hospital realized a new orthodontic device to preserve oral and perioral tissue of LNS patients. At first irreversible hydrocolloid impressions of maxillary and mandibular arches were obtained to realize individual impression trays.

**Figure 1:**
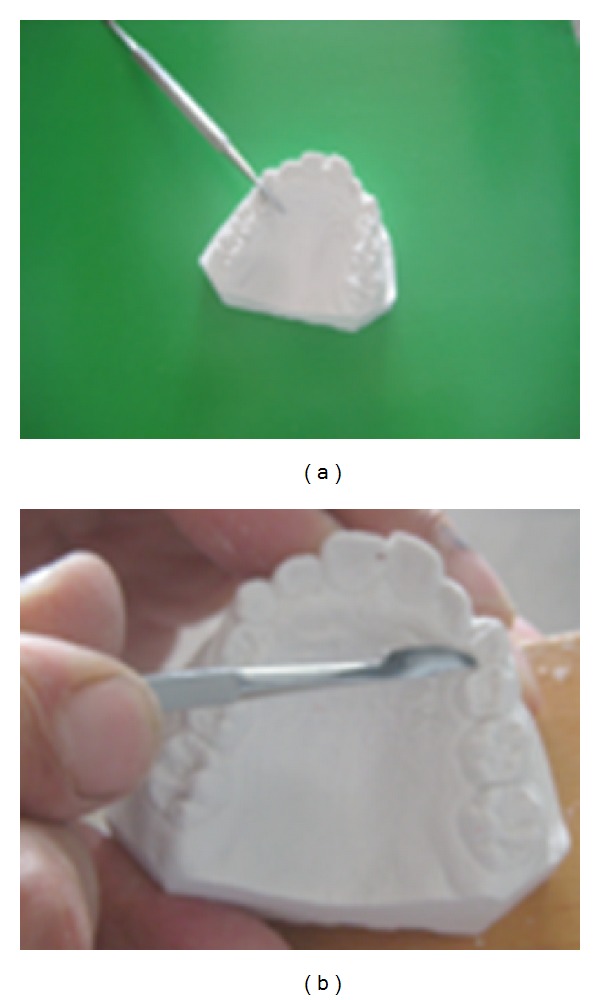
Plaster model and elimination of undercuts.

**Figure 2:**
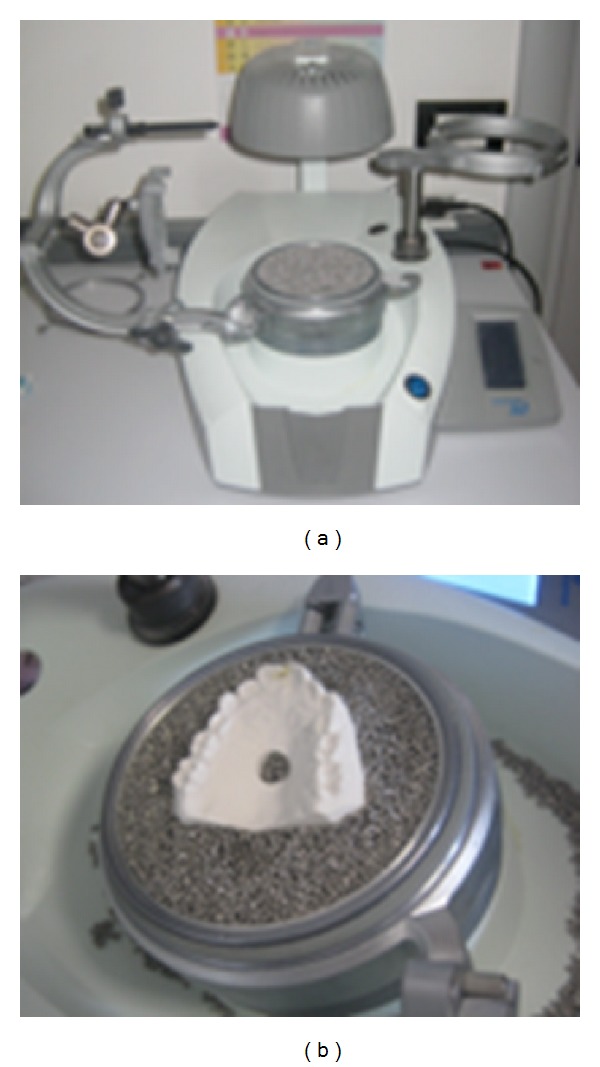
Plaster model in the positive pressure thermoformer.

**Figure 3:**
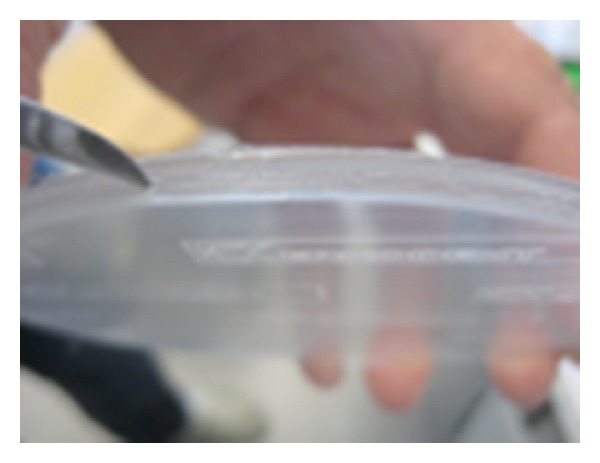
Thermoforming disc, the double matrix is high-lighted.

Second impressions were made with individual trays and an occlusal wax recorded the occlusion. These impressions were finally taken in spite of patient's lack of compliance; operators' persuasive capacity, family, and operators' efforts to change the negative behavior of M.C. were crucial. On these impressions a plaster model was built, the undercuts were eliminated (Figures 1 and 2), and the orthodontic dispositive was realized by thermoforming disc materials (with a hard copolyster outer layer and a soft polyurethane inner layer) (Figure 3), using a positive-pressure thermoformer (Ministar) (Figures 2 and 5).

**Figure 4:**
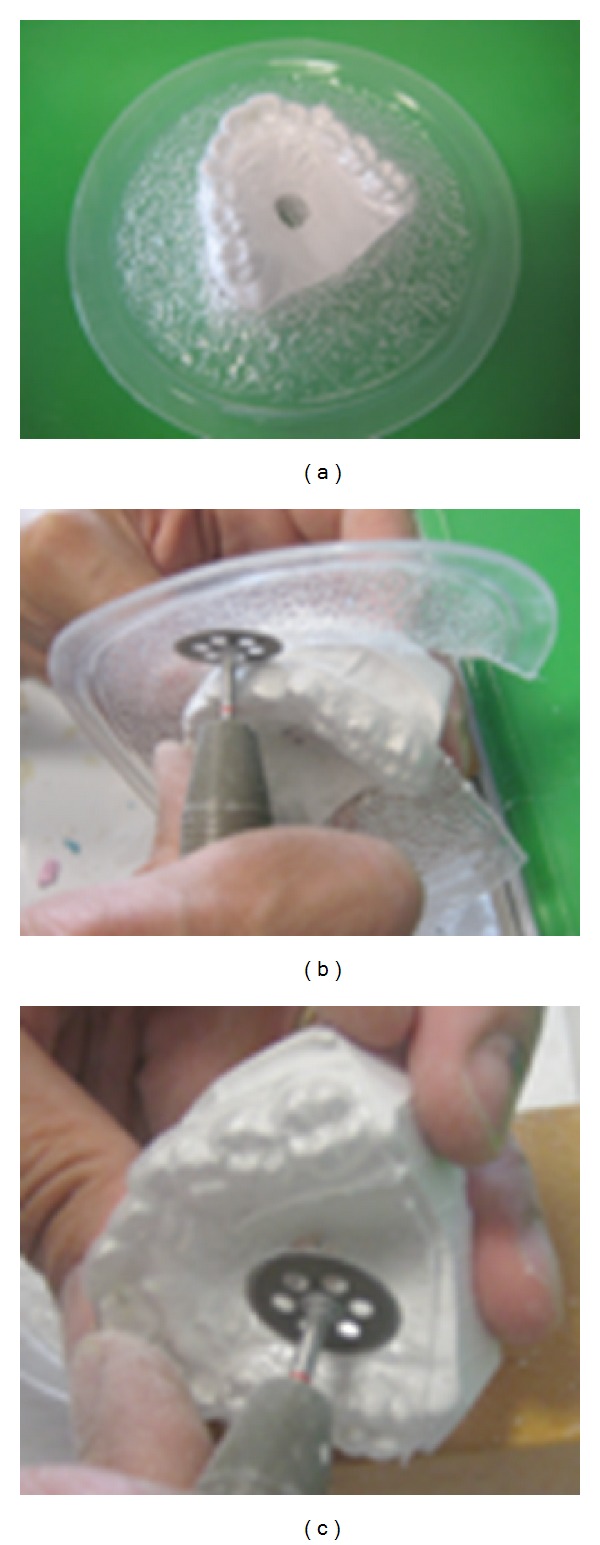
The appliance is cut out and the mucosal flange is reduced.

**Figure 5:**
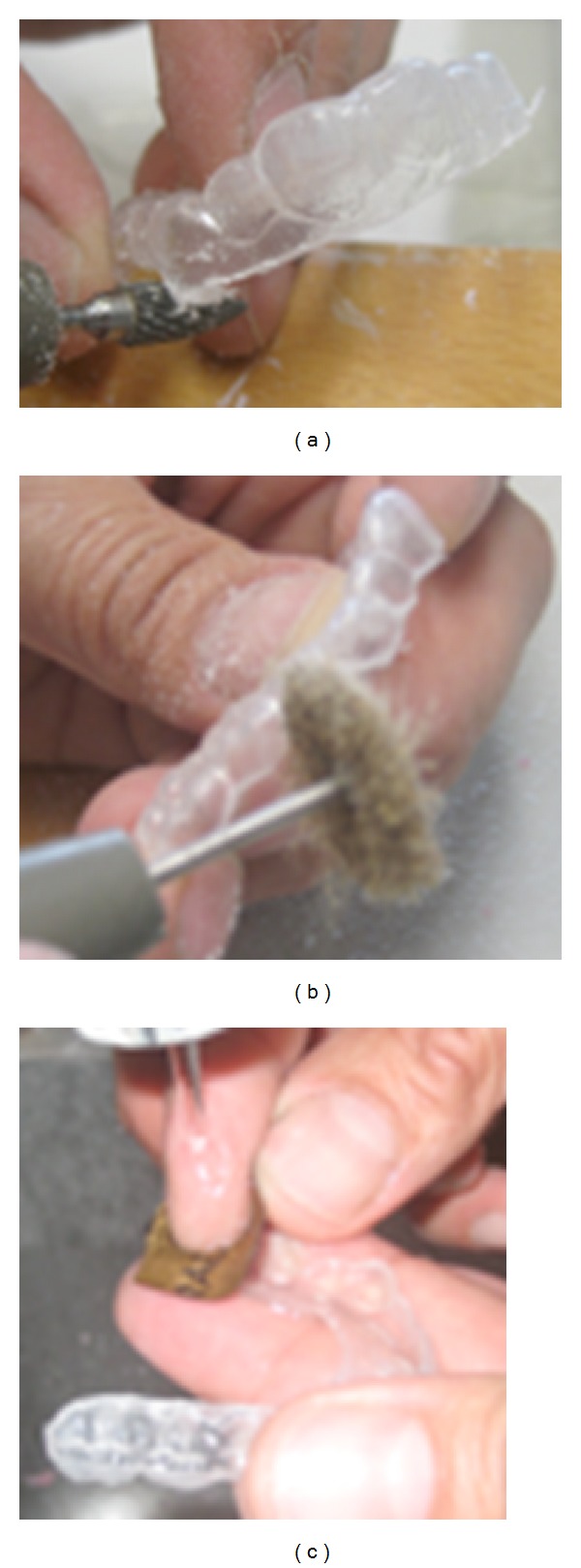
The resin mouthguard is well refined.

The hard side was placed in contact with teeth to increase friction and retention; on the contrary the soft one was placed in contact with antagonist dental arch to prevent traumatic occlusion. The first devices were removed by patients' tongue, so it was decided to change the dispositive, reducing mucosal flange to prevent the mouthguard's removal (Figure 4).

At the beginning, patients used the device only for few hours, but time by time they were persuaded to wear it longer. At first a thick lower bite was made to produce an anterior open bite; then it was substituted by a thinner maxillary and mandibular mouthguards well tolerated by the patient also during the meals.

After these treatments, the patients were able to wear the device for 24 hours and it guaranteed the health of the oral and perioral tissue.

## 4. Discussion

The most common disadvantages of preventive intraoral devices are the design that may cause fungal infections, extensive laboratory time, and difficulty in daily oral care. Also frequent adjustments are required and heavy biting forces added to poor cooperation may interfere with impression taking.

These devices extend to the vestibule area and the splint that creates an open bite increases the drooling habit with a consequent fungal infection of chin dermis. Cemented splint requires an extensive laboratory work, daily hygiene care is difficult, and the demineralization of the teeth is frequent.

The presence of metal clasps can make the placement of device more difficult. The intraoral impression is necessary to create a device customized for each individual case.

Some patients with LNS show a degree of collaboration to allow the intraoral alginate impression, in other LNS patients is impossible to have a degree of cooperation on making the intraoral impression; in fact sedation is sometime necessary.

The use of putty-type vinyl polysiloxane impression material or quick alginate to overcome the insufficient patient's collaboration that makes the intraoral impression very difficult is suggested. It is possible to obtain greater patient's collaboration thanks to behavioral modification and persuasive capacity of family and operators.

The simple intraoral device presented by Gaslini operators is easy to fabricate; it is a mouthguard realized by thermoforming disc and characterized by two different matrices: hard and soft. The hard matrix guarantees a good retention, so during the cementation, the head straps, the splint extension to vestibule area, and the metal clasps are not necessary to give stability. The soft matrix protects the oral and perioral tissue from traumatic bite. The double matrix device is readily removed for everyday cleaning and the placement is also comfortable. The bite is very thin, it permits that the patient can wear the mouthguard all day, also during the meals, and consequently the drooling habit is not accentuated. The device is built on the patient plaster model; it can be customized for each individual case. Heavy biting force and poor patient's collaboration may interfere with impression taking.

In the case described here the first impression to create an individual impression trays was taken and a second one to fabricate the device.

It was possible because the patient was collaborative; he modified his behaviour. Also the work was simplified by the use of quick alginate.

In a second moment, the mucosal flange was reduced to prevent dispositive removal caused by the tongue.

The efficacy of this device was observed during a period of about 7 years. During these years self-inflicted oral and/or perioral mutilations did not happen. The major difficulties encountered during this therapy are as follows:

- necessity of modifying the dispositive to adapt it to the mouth changes,
- risk of small cracks on the occlusal resin bite that must be often repaired.

## 5. Conclusions

In conclusion the literature analysis demonstrates that the intraoral appliances, despite some limitations, can represent a good alternative option to invasive treatment and a good choice of therapy to limit and to prevent self-mutilative behavior in LNS patients.

The thin resin mouthguard described was successfully employed to prevent self-mutilation of oral and perioral structures. It represented a conservative solution and a good alternative option to invasive treatment of extracting the teeth or of orthognathic surgery.

In addition, the device is easy to build and is well accepted by patient. A good choice of treatment for improving the quality of life of these patients can be represented.
